# Supplementary figures and images for: Genome-Wide Characterization of the Phenylalanine Ammonia-Lyase Gene Family and Their Potential Roles in Response to Aspergillus flavus L. Infection in Cultivated Peanut (Arachis hypogaea L.)
Source: Genes (Basel). 2024 Feb 21;15(3):265. doi: 10.3390/genes15030265 (PMC10970321; doi:10.3390/genes15030265)

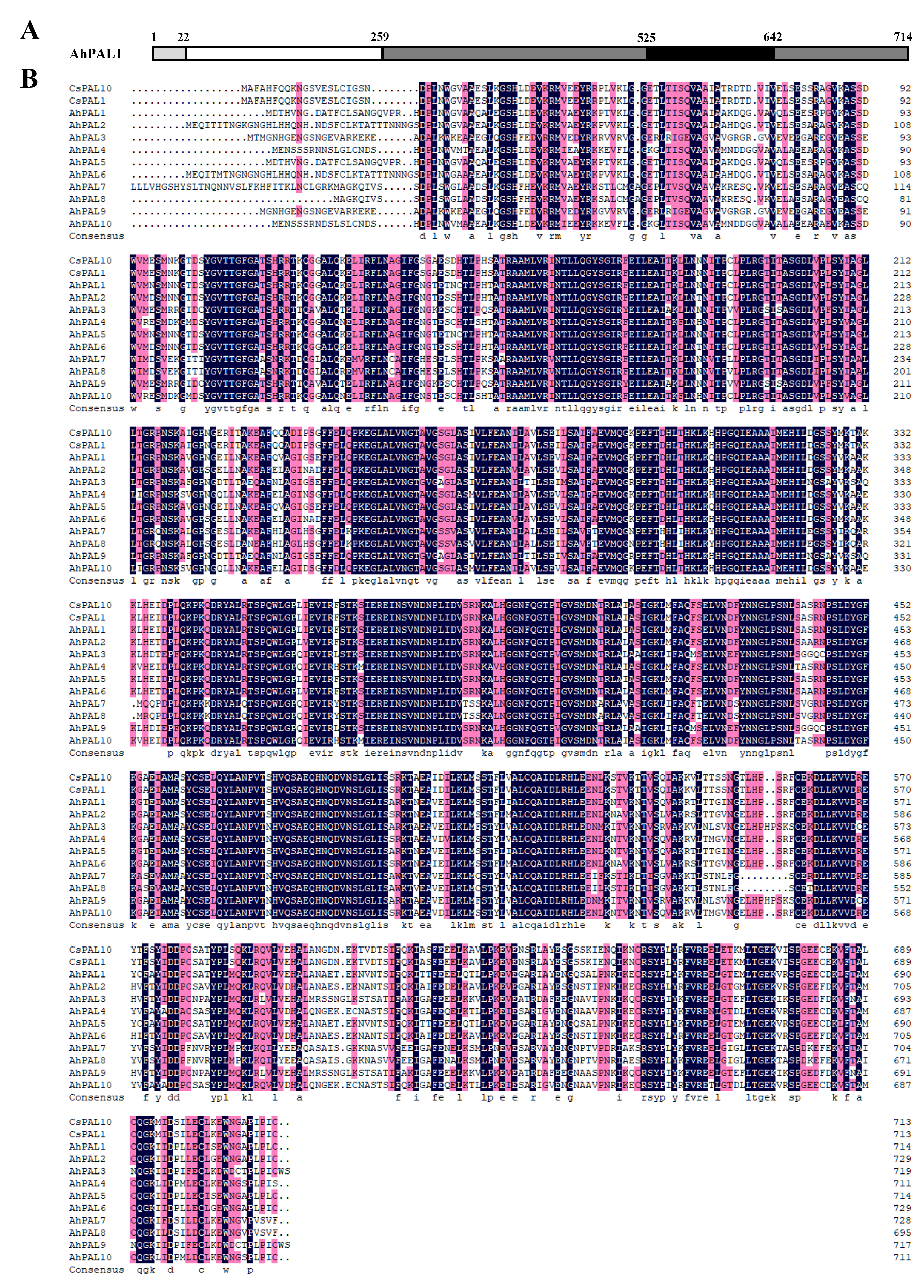

Supplement: Supplementary file 1 [file genes-15-00265-s001.zip › Figure S1.tif]

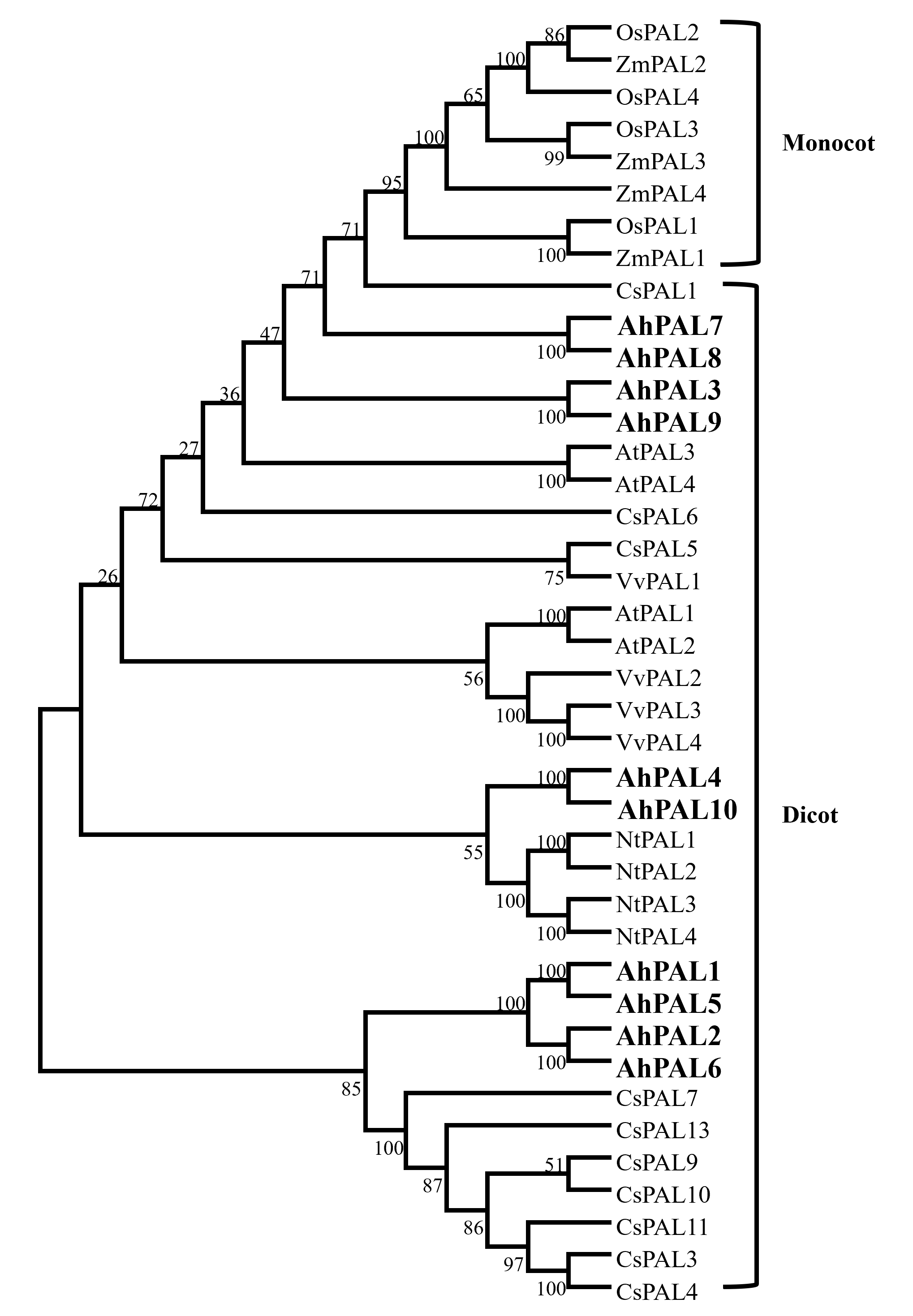

Supplement: Supplementary file 1 [file genes-15-00265-s001.zip › Figure S2.tif]

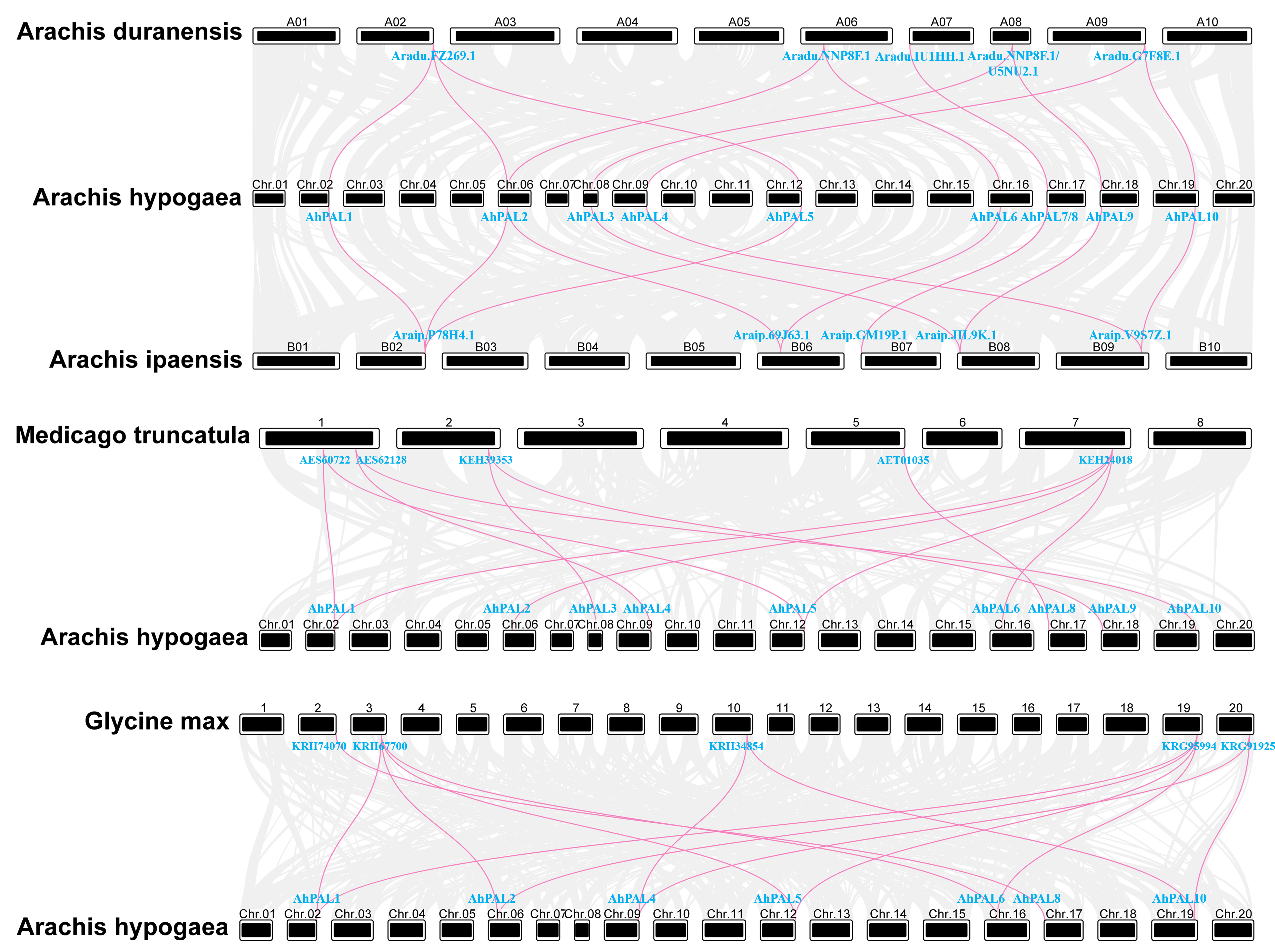

Supplement: Supplementary file 1 [file genes-15-00265-s001.zip › Figure S3.tif]

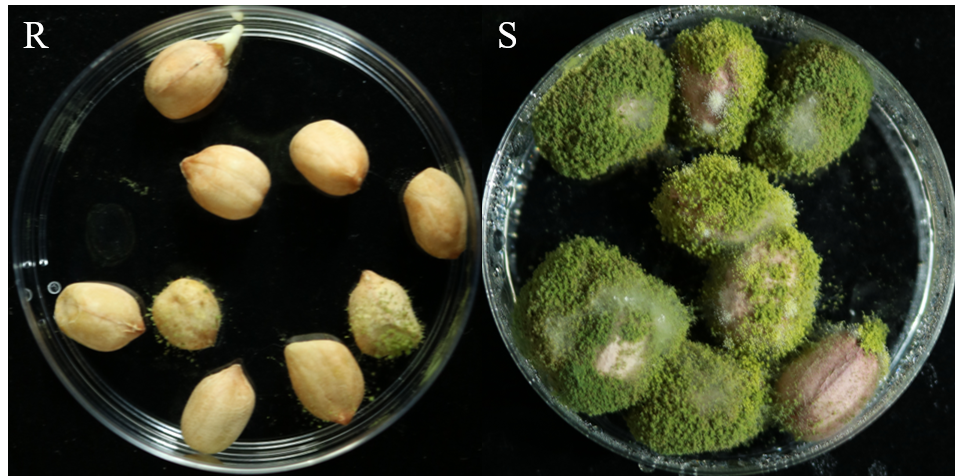

Supplement: Supplementary file 1 [file genes-15-00265-s001.zip › Figure S4.tif]
